# The Relationship between Plasma Soluble Receptor for Advanced Glycation End Products and Coronary Artery Disease

**DOI:** 10.1155/2019/4528382

**Published:** 2019-06-02

**Authors:** Xiangming Wang, Tingting Xu, Deeraj Mungun, Chuanwei Zhou, Zhimin Zha, Miao Lu, Chuyan Fen, Yan Guo

**Affiliations:** Department of Geriatric Cardiology, The First Affiliated Hospital of Nanjing Medical University, Nanjing, China

## Abstract

**Background:**

Inflammation is involved in the development and progression of coronary artery disease (CAD). The role of the receptor for advanced glycation end products (RAGE) in the development of CAD has been recognized. The expression of sRAGE and S100A12 in patients with coronary artery disease from different studies was inconsistent. We attempted to determine the expression of sRAGE and S100A12 and their relationship in the subjects with different severity levels of CAD.

**Methods:**

A total of 259 patients undergoing coronary angiography were enrolled from the Department of Geriatric Cardiology in the First Affiliated Hospital of Nanjing Medical University from January 2014 to December 2016. Groups were divided as follows: normal coronary artery (control group), nonobstructive coronary atherosclerosis (<50% stenosis in all coronary vessels, NOCA group), stable angina (SAP group), and acute coronary syndrome (ACS group). During CAG or PCI, peripheral arterial blood was collected from all the patients. Plasma sRAGE and S100A12 levels were measured by ELISA. We calculated the SYNTAX score of each patient with CAD according to the result of CAG.

**Results:**

The levels of sRAGE were significantly elevated in the ACS group compared with those in the control group, the NOCA group, and the SAP group. sRAGE levels were similar among the control group, the NOCA group, and the SAP group. Plasma S100A12 levels were significantly higher in the ACS group than in the control group and the NOCA group. Baseline correlations between plasma levels of sRAGE and plasma S100A12 in the ACS group were significant. Plasma sRAGE concentration was increasing in patients with ACS and was significantly positively correlated with the increasing SYNTAX score. ROC curve analysis revealed that the combination of sRAGE and S100A12 had a good performance in the prediction of high-risk CAD patients.

**Conclusion:**

The plasma levels of sRAGE and S100A12 can be increased in patients with ACS. The elevated sRAGE concentration may be independently associated with the severity of CAD and the inflammatory process. sRAGE combined with S100A12 may be used as a predictor of severe coronary heart disease.

## 1. Introduction

The pathogenesis of coronary artery disease (CAD) is complicated and has not yet been fully elucidated. Numerous studies have found that inflammation is involved in the development and progression of coronary artery disease [1]. And the role of the receptor for advanced glycation end products (RAGE) in the development of CAD has been recognized [2, 3].

RAGE, a transmembrane receptor of the immunoglobulin superfamily [4], expressed in a wide variety of cells including endothelial cells, monocytes, and vascular smooth muscle cells [5], has three isoforms that include full-length RAGE, N-truncated RAGE, and C-truncated RAGE [6]. RAGE, a multiligand receptor, interacts with a variety of ligands including the AGEs, S100 calcium-binding protein family, high-mobility group box 1 (HMGB1), beta amyloid-like peptides, and *β*2 integrins [2, 5, 7]. The binding of RAGE with its ligands results in amplifying the inflammatory response and tissue injury via the activation of nuclear factor kappa-B, the release of cytokines, the expression of adhesion molecules, and the generation of reactive oxygen species, contributing to the pathogenesis of atherosclerosis and plaque rupture [8–11].

The S100 calcium-binding protein family has at least 25 members [12] including S100B, S100P, S100Z, S100G, Repetin, and the multidomain proteins trichohyalin, filaggrin, and S100A1-A18 in which S100A12, also termed extracellular newly identified RAGE-binding protein (EN-RAGE), expressed in white blood cells, is an inflammation mediator [13–15]. Previous studies have shown that the ligation of S100A12 to RAGE leads to inflammation [13, 15]. Thus, CAD patients have a higher plasma S100A12 concentration than healthy controls [9, 16, 17], and S100A12 showed a concentration-dependent increase in the long-term major adverse cardiac and cerebrovascular event (MACCE) rate in stable CAD patients [13].

The C-truncated RAGE, a soluble fragment, is called the human soluble receptor for advanced glycation end products (sRAGE). Due to the ability to bind to AGEs and the lack of intracellular signal transduction, sRAGE prevents the activation of full-length RAGE [18, 19] and decreases inflammatory injury. It is hypothesized that high sRAGE concentrations were proportional to the severity of CAD, thereby exerting an antiatherosclerotic effect. However, current data are diverging. Falcone et al. [15] reported that plasma sRAGE levels were significantly lower in acute coronary syndrome (ACS) patients than in stable angina patients. However, Basta et al. [18] found that sRAGE levels did not differ between the NSTE-ACS group and the stable angina group. McNair et al. [11] showed that serum sRAGE levels were lower in NSTEMI patients than in healthy control subjects and inversely related to the number of diseased vessels.

Few studies have evaluated the effect of diabetes on the sRAGE and S100A12 levels in patients with CAD. Previous reports have demonstrated that sRAGE levels were significantly higher in diabetic patients than in nondiabetic subjects, and CAD patients with diabetes have higher sRAGE levels than non-CAD patients with diabetes [19]. The study by Zhao et al. [16] reported that serum S100A12 levels were significantly higher in patients with T2DM with CAD than in those without CAD.

Taken together, the expression of sRAGE and S100A12 in patients with coronary artery disease is controverting. Therefore, in the present study, we attempted to determine the expression of sRAGE and S100A12 and their relationship in the subjects with different severity levels of CAD, and we also attempted to evaluate what influence diabetes has on them.

## 2. Materials and Methods

### 2.1. Study Population

The study included patients undergoing coronary angiography who were hospitalized from January 2014 to December 2016 in the Department of Geriatric Cardiology at the First Affiliated Hospital of Nanjing Medical University. The patients were ≥18 years old and grouped based on coronary angiography and their clinical symptoms. The following are the exclusion criteria: (1) patients with severe cardiac insufficiency (LVEF ≤ 30%); (2) serious comorbidities, liver failure, renal failure, connective tissue disease, and oncology and infectious diseases; (3) patients with acute hemorrhagic disease; and (4) acute stroke patients. A total of 259 patients were enrolled, including 75 patients with acute coronary syndrome (ACS group) consisting of 53 males and 22 females aged 48-87 (66.12 ± 9.69) years. In the stable angina pectoris group (SAP group), there were 57 patients, including 44 males and 13 females, aged 45-83 (64.51 ± 9.25) years. 64 patients with <50% stenosis in all coronary vessels were classified as the nonobstructive coronary atherosclerosis (NOCA) group, including 42 males and 22 females, aged 46-80 (63.83 ± 7.85) years. 63 patients with no stenosis in coronary arteries, including 33 males and 10 females, were in the control group, ranging from 31 to 76 (57.30 ± 8.64) years. The study protocol conformed to the guidelines of the Helsinki Declaration for human research and was approved by our local ethics committee.

### 2.2. Clinical Data Collection and Biochemical Factor Measurements

Clinical data and the basic information of all patients, including gender; age; height; body mass index (BMI); hypertension, diabetes, and other previous medical history; systolic blood pressure; diastolic blood pressure; and heart rate were collected. Fasting blood glucose, total cholesterol, low-density lipoprotein, high-density lipoprotein, triglyceride, serum creatinine, uric acid, liver function, and renal function were measured by an automatic biochemical analyzer.

### 2.3. Coronary Angiography

Each study was performed using GE Innova 3000 angiography CAG examination, through a radial artery or femoral artery puncture, using selective JL and JR angiography catheters, respectively, into the left and right coronary artery openings with multibody shot; the results of coronary angiography were then recorded. Coronary heart disease was defined as ≧50% stenosis in major vessels (left anterior descending artery, circumflex artery, and right coronary artery and their major branches).

### 2.4. SYNTAX Point Calculation

Two specialized physicians who have long been involved in coronary intervention evaluated the angiographic findings and performed a comprehensive evaluation of the left main coronary artery, left anterior descending branch, left circumflex artery, and right coronary artery stenosis. Each lesion was scored one by one using the online SYNTAX scoring system (http://www.syntax.score.com) according to the anatomical and pathological features, such as the location of the lesion, the degree of stenosis, the degree of bifurcation, the degree of calcification, and whether it was diffuse; then, the score was added to the patient's SYNTAX score. Patients with scores ranging from 1 to 22 comprised the low-risk group, those with 23 to 32 comprised the moderate-risk group, and those with ≥33 comprised the high-risk group.

### 2.5. Sample Collection and Processing

After the puncture was successfully implanted into the arterial sheath, 5 ml of radial artery or femoral artery blood (peripheral blood) was drawn. All blood was centrifuged at 3000 r/min for 10 min at room temperature; then, 0.5 ml of plasma was taken and kept frozen at -70°C.

### 2.6. Plasma Cytokine Assay

Plasma sRAGE and S100A12 levels were measured using the sRAGE S100A12 ELISA Kit (Nanjing SenBeiJia Biological Technology Co. Ltd.). According to the instructions, the OD values of each plasma sample and standard were determined according to the instructions and the standard curve was drawn. Based on the OD value of each sample, the sample concentration was calculated (interassay CV < 8%, intra − assay CV < 10%; sensitivity range: sRAGE 31.2-2000 pg/m and S100A12 0.5 ng/ml-50 ng/ml).

### 2.7. Statistical Analysis

Data analysis was accomplished using SPSS 21.0 software. The measurement data were expressed as$\bar{x}\pm s$. Student's*t*-test was used for comparison between two groups, and ANOVA analysis was used for comparison among multiple groups. The frequency was used for counting data, and the *χ*^2^ test was used for comparison among groups. Pearson's correlation analysis was used for single factor and logistic regression analysis. The correlation analysis of multiple factors was performed. The receiver operating characteristic (ROC) curve was used to determine the predictive values of sRAGE and S100A12 on severe coronary heart disease. *P* < 0.05 for the difference was statistically significant.

## 3. Results

### 3.1. Baseline Characteristics

The baseline characteristics of the subjects are presented in Table 1. Those who developed CAD were older, had a higher prevalence of diabetes, and had a higher blood pressure (*P* < 0.05). CAD patients were more likely to take clopidogrel and statins (*P* < 0.05). The use of other medications did not differ among groups. No significant differences were observed among all groups regarding male gender, hypertension, history of smoking, BMI, HR, HGB, FBS, and the serum levels of TC, LDL-C, TG, SUA, and SCR (as shown in Table 1).

### 3.2. Plasma sRAGE Levels in Different Groups

**Figure 1:**
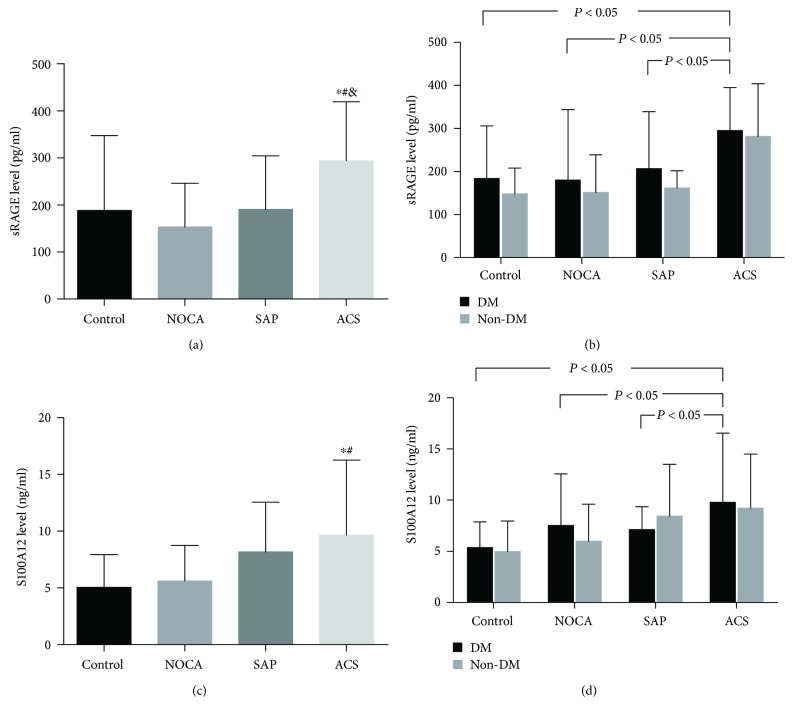
Plasma sRAGE and S100A12 levels in different groups. (a) Plasma levels of sRAGE in four groups. (b) Plasma levels of sRAGE in four groups with or without DM. (c) Plasma levels of S100A12 in four groups. (d) Plasma levels of S100A12 in four groups with or without DM.

The levels of sRAGE were significantly elevated in the ACS group compared with the other groups. No statistically significant differences were shown among the control group, the NOCA group, and the SAP group (293.65 ± 125.77 ng/ml in the ACS group, 190.64 ± 113.86 ng/ml in the SAP group, 153.52 ± 92.64 ng/ml in the NOCA group, and 188.01 ± 159.65 ng/ml in the control group; ACS vs. control, *P* < 0.01; SAP vs. control, *P* = 0.916; NOCA vs. control, *P* = 0.151; ACS vs. SAP, *P* < 0.01; ACS vs. NOCA, *P* < 0.01; and NOCA vs. SAP, *P* = 0.053) (Figure 1(a)).

Different groups of sRAGE levels in DM patients were higher than those in non-DM patients, but there was no statistical difference (control: 183.87 ± 122.14 ng/ml vs. 148.28 ± 59.96 ng/ml, *P* > 0.05; NOCA: 151.48 ± 87.40 ng/ml vs. 180.11 ± 164.00 ng/ml, *P* > 0.05; SAP: 207.01 ± 131.75 ng/ml vs. 161.80 ± 40.26 ng/ml, *P* > 0.05; and ACS : 295.33 ± 99.95 ng/ml vs. 281.25 ± 122.78 ng/ml, *P* > 0.05). However, in DM patients, the sRAGE levels of the ACS group were significantly higher (*P* < 0.05) compared to those of the other groups (Figure 1(b)).

### 3.3. Plasma S100A12 Levels in Different Groups

S100A12 levels were increased only in the ACS group compared to the control group, and no significant difference was found among the control group, the NOCA group, and the SAP group (9.66 ± 6.59 ng/ml in the ACS group, 8.17 ± 4.38 ng/ml in the SAP group, 5.06 ± 2.89 ng/ml in the NOCA group, and 5.06 ± 2.89 ng/ml in the control group; ACS vs. control, *P* = 0.023; NOCA vs. control, *P* = 0.142; SAP vs. control, *P* = 0.585; and NOCA vs. SAP, *P* = 0.246) (Figure 1(c)). S100A12 levels in the ACS group were significantly higher than those in the control group and the NOCA group, but no difference was comparable to the SAP group (ACS vs. control, *P* = 0.023; ACS vs. NOCA, *P* = 0.031; and ACS vs. SAP, *P* = 0.599) (Figure 1(c)).

As shown in Figure 1(d), stratifying for all groups by DM, diabetes in the ACS group had higher plasma s100A12 levels than the other groups (DM patients: control, 5.36 ± 2.52 ng/ml; NOCA, 7.53 ± 5.05 ng/ml; SAP, 7.12 ± 2.24 ng/ml; and ACS, 9.79 ± 6.77 ng/ml; *P* < 0.05). Plasma s100A12 levels were comparable between DM and non-DM patients in all groups (control: 5.36 ± 2.52 ng/ml vs. 4.97 ± 2.98 ng/ml; NOCA: 7.53 ± 5.05 ng/ml vs. 6.00 ± 3.61 ng/ml; SAP: 7.12 ± 2.24 ng/ml vs. 8.44 ± 5.07 ng/ml; and ACS: 9.79 ± 6.77 ng/ml vs. 9.22 ± 5.29 ng/ml; DM vs. non-DM in all groups, *P* > 0.05).

### 3.4. Association between sRAGE and S100A12

**Figure 2:**
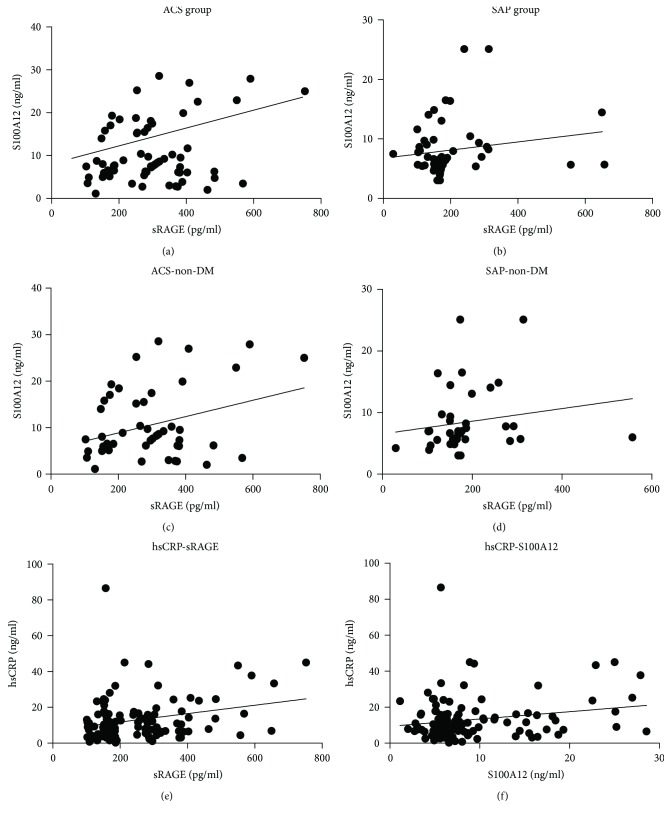
(a–d) Association between sRAGE and S100A12 in the ACS group. (e) Association between sRAGE and hsCRP in CAD patients. (f) Association between S100A12 and hsCRP in CAD patients.

Baseline correlations between plasma levels of sRAGE and plasma S100A12 in the ACS group were significant (Pearson *r* = 0.281, *P* = 0.015) (Figure 2(a)). In contrast, there was no correlation between sRAGE and S100A12 among the SAP patients (Pearson *r* = 0.176, *P* = 0.190) (Figure 2(b)). We studied the relationship between the plasma levels of sRAGE and S100A12 in nondiabetic CAD patients. The correlations between the plasma levels of sRAGE and s100A12 in the nondiabetic ACS group were significant (Pearson *r* = 0.327, *P* = 0.0192) (Figure 2(c)). In contrast, there was no correlation between sRAGE and s100A12 in nondiabetic SAP patients (Pearson *r* = 0.122, *P* = 0.443) (Figure 2(d)). In CHD patients, sRAGE and hsCRP were significantly positively correlated (*r* = 0.280, *P* = 0.001) (Figure 2(e)) and S100A12 was also positively correlated with hsCRP (*r* = 0.206, *P* = 0.20) (Figure 2(f)).

### 3.5. Correlation between Plasma Levels of sRAGE and SYNTAX Score (SS)

**Figure 3:**
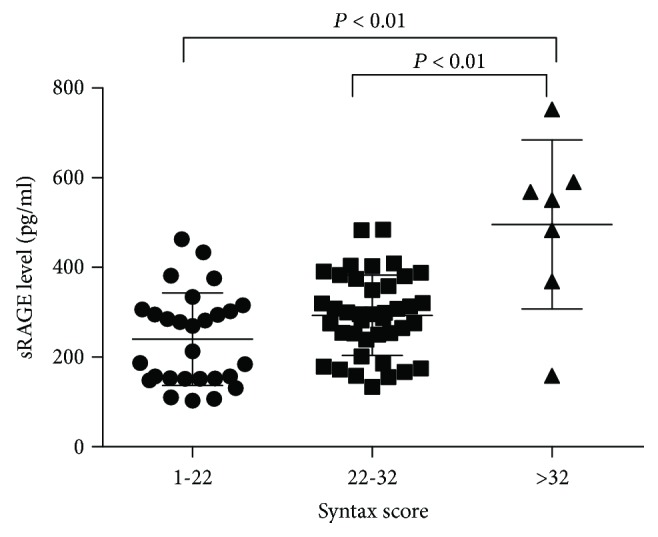
Plasma sRAGE levels in different risk groups by SYNTAX score.

**Figure 4:**
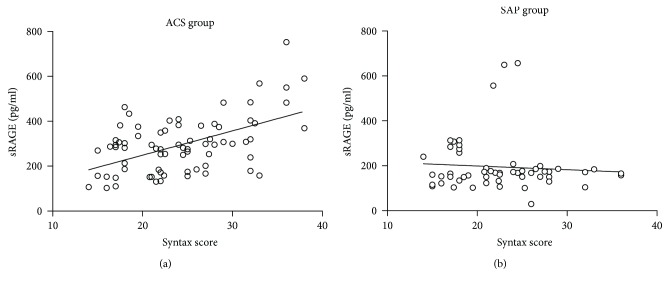
(a) Correlation between plasma levels of sRAGE and SYNTAX score in the ACS group. (b) Correlation between plasma levels of sRAGE and SYNTAX score in the SAP group.

Patients were divided into three tertiles according to their SYNTAX score as follows: low-risk group, SS ≥ 22; moderate-risk group, 22 ≤ SS < 33; and high-risk group, SS ≥ 33. In the ACS group, the plasma sRAGE concentration in the high-risk group was significantly higher than that in the moderate-risk group and the low-risk group (Figure 3). The plasma levels of sRAGE were significantly positively correlated with the SYNTAX score tertiles in the ACS group (Pearson *r* = 0.535, *P* < 0.01) (Figure 4(a)). Conversely, there is no significant difference in the correlation between plasma sRAGE and SYNTAX score in the SAP group (Pearson *r* = 0.076, *P* = 0.574) (Figure 4(b)).

### 3.6. Comparison of Receiver Operating Characteristic (ROC) Curves for sRAGE, S100A12, and the Combination of sRAGE and S100A12 in the Prediction of Higher SS

**Figure 5:**
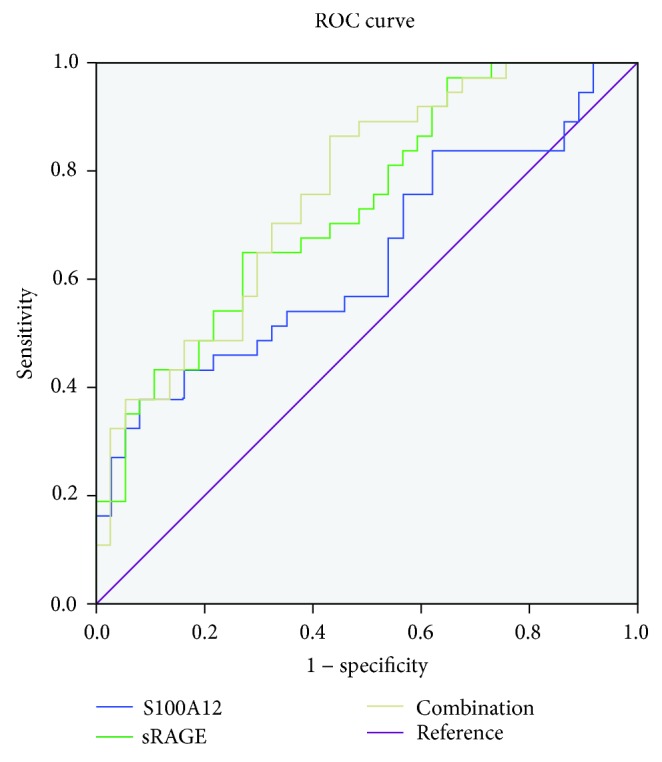
ROC curves for sRAGE, S100A12, and the combination of sRAGE and S100A12 in the prediction of higher SYNTAX score (SS).

The area under the ROC curves for sRAGE was 0.734 (95% CI: 0.622-0.864, *P* = 0.001) in the prediction of the severity of CAD patients (SS ≥ 33). The area under the curve (AUC) for S100A12 was 0.637 (95% CI: 0.509-0.764, *P* = 0.043). To further evaluate the diagnostic value of the novel logistic regression risk prediction model, the predictive probability of a high SYNTAX score was calculated by the combination of sRAGE and S100A12 for each patient and then subjected to ROC analysis. By combining these independent factors, the AUC was increased to 0.795 (95% CI: 0.651-0.867, *P* < 0.001) (Figure 5).

## 4. Discussion

In our present study, we attempted to determine the expression of sRAGE and s100A12 and their relationship in the subjects with different severity levels of CAD. We further evaluated the effect of diabetes on the sRAGE and s100A12 levels in patients with CAD. We performed a cross-sectional study and detected plasma sRAGE and s100A12 levels in different CAD groups. Our results showed that plasma sRAGE and s100A12 levels significantly increased only in patients with ACS. Stratifying for all groups by DM, plasma sRAGE and s100A12 levels were comparable between DM and non-DM patients in all groups. We calculated the SYNTAX score of each ACS patient and found that the elevated sRAGE concentration may be independently associated with the severity of CAD and the inflammatory process. Using ROC curve to determine the predictive values of sRAGE and s100A12 on severe coronary heart disease, we found that sRAGE combined with S100A12 may be used as a predictor of severe coronary heart disease.

A number of studies have observed the expression of sRAGE in patients with acute myocardial infarction. Basta et al. [18] showed that sRAGE was significantly higher in patients with acute myocardial infarction than in patients with stable angina and was positively correlated with troponin I. Wang et al. [20] found that sRAGE in the myocardial infarction group was significantly higher than that in the control group. Cai et al. [21] showed that the level of sRAGE in the acute myocardial infarction group was significantly higher than that in the noninfarcted group. However, patients with non-ST elevation myocardial infarction did not have elevated sRAGE due to the weaker inflammatory response. McNair et al. [11, 22] found that plasma sRAGE levels were decreased in patients with non-ST-segment elevation myocardial infarction, while the levels of TNF-*α* and hypersensitive C-reactive protein increased. Whether in the control group or the infarction group, the level of sRAGE was negatively correlated with TNF-*α* and high-sensitivity C-reactive protein. In addition, Jensen et al. [23] proposed that the time point of blood sample collection is important, and they reported that sRAGE levels were high in the early phase rather than in the days after AMI and primary percutaneous coronary intervention (pPCI) in STEMI patients. The role of sRAGE as a protective factor in acute myocardial infarction remains to be further studied.

sRAGE changes in different types of coronary heart disease are more complicated, and the existing research data is still controversial [24]. Therefore, this study further compared ACS, stable angina pectoris, and nonstenosis coronary atherosclerosis in patients with sRAGE expression, using the normal group as a control. It was found that the average sRAGE and S100A12 levels are significantly higher in patients with acute coronary syndrome than in the control group. The reason may be because plaque instability, plaque rupture, and inflammation are involved in acute coronary syndrome. In acute coronary events, the binding of advanced glycation end product receptors and their ligands (S100, HMGB1, AGEs, etc.) increases the inflammatory mediator levels and oxidative stress, thereby aggravating myocardial injury [25]. sRAGE may serve as a protective factor for the body. When released into the blood, sRAGE inhibits the binding of RAGE to its ligands, reducing inflammation in myocardial compensatory protection. In addition, in patients with ACS, the level of sRAGE is positively correlated with the level of S100A12 and positively correlated with the severity of the coronary artery, which further indicates that sRAGE may be used as a new serological marker to evaluate the degree of inflammatory response and the severity of ACS.

Current research shows that sRAGE levels were elevated in patients with ACS and in patients with diabetes. In contrast, sRAGE levels were lower in stable CAD patients without diabetes but were elevated in diabetic patients. Falcone et al. [26] found that patients with CAD without diabetes had lower sRAGE than normal controls. However, in diabetic CAD patients, Colhoun et al. [10] found that their sRAGE levels were higher than normal. In our study, the patients were divided into diabetic and nondiabetic subgroups, and the results showed that in different types of coronary heart disease patients, the plasma levels of sRAGE and S100A12 were all increased in patients with diabetes mellitus compared with nondiabetic patients. Patients in the ACS group had higher sRAGE than the control group and the stable angina pectoris group with or without diabetes mellitus. However, patients with stable CAD with diabetes mellitus had an increased level of sRAGE compared with the control group, but they failed to show statistical difference. There was no significant difference in plasma sRAGE between nondiabetic patients with stable CAD and the control group. The reason for the failure to show a statistically significant difference in stable CAD may be related to the small size of the study.

However, in patients with stable angina and atherosclerosis, we observed the opposite result. Atherosclerosis is a chronic inflammatory process that leads to the onset of clinical events by inducing the development of atherosclerotic plaques and subsequent thrombosis. Endothelial dysfunction plays an important role in the initiation and progression of atherosclerosis. RAGE is expressed in a variety of cells including endothelial cells. Numerous studies have shown that AGE and its receptor (RAGE) system is associated with atherosclerosis and restenosis [27, 28]. The binding of AGE to RAGE changes intracellular signal transduction, mediates inflammation, and induces atherosclerosis [27]. Tam et al. [8] found that the level of serum sRAGE in diabetic patients was decreased, while the expression of RAGE on monocytes was increased. Therefore, sRAGE is one of the most important molecules involved in the development of atherosclerosis. sRAGE is considered as a protective factor against atherosclerosis, especially in diabetic patients. Low plasma sRAGE as an independent risk factor is also associated with coronary heart disease in nondiabetic patients [29]. Our study suggests that in patients with stable angina and nonstenosis of atherosclerosis, plasma sRAGE concentrations were decreased compared with the control group, but the difference was not statistically significant. Atherosclerosis is often a chronic inflammatory process with less severe inflammatory reactions than ACS. The release of sRAGE often acts as a response to atherosclerosis in the body. Furthermore, sRAGE can competitively bind with ligands of RAGE to reduce inflammation and delay the progression of atherosclerosis. Low-level plasma sRAGE and the development of atherosclerosis are closely related. We hypothesize that low sRAGE levels in stable CAD may reflect the release of local RAGE from atherosclerotic blood vessels. sRAGE may capture RAGE ligands, thereby reducing circulating measurable sRAGE and further reducing the activity of the RAGE axis.

Previous studies have shown that S100 protein family members are involved in cardiovascular disease, such as S100B, S100A8, S100A9, and S100A12 [21, 30, 31]. S100A12, released by glial cells, oligodendrocytes, and other neural tissue cells, is secreted intoCID="C036" value="to" the extracellular space and mediates biological functions. S100 activates RAGE-mediated inflammatory pathways and increases the expression of adhesion molecules and inflammatory cytokines, leading to atherosclerosis [32]. Myocardial ischemia or necrosis promotes the production of inflammatory factors such as S100B, S100A6, S100P, and RAGE in the infarct area; activates the S100-RAGE axis; and induces the increase of inflammatory cytokines. The release of a large number of S100 proteins and other inflammatory factors exacerbates myocardial damage, apoptosis, and myocardial remodeling, creating a vicious cycle that leads to cardiac dysfunction. This study found that S100A12 levels in patients with ACS and stable angina pectoris were higher than those of the control group. S100A12, an inflammatory marker, was significantly elevated in ACS, consistent with previous findings.

This study further analyzes the correlation between plasma s100A12 and sRAGE. As for the association between sRAGE and s100A12, sRAGE prevents the interaction of s100A12 with RAGE, decreasing the activation of RAGE. If this mechanism is dominant, this association would be positive, relating to fewer vascular events. However, according to a study by Kim et al. [29], plasma s100A12 was positively correlated with vascular calcification, whereas sRAGE was negatively correlated with it. In one report on haemodialysis (HD) patients, sRAGE showed a negatively independent association with vascular calcification scores (VCS), but S100A12 showed no association with VCS [33]. In a study with 100 nondiabetic patients with premature CAD, S100A12 levels were increased and sRAGE revealed a negative association with s100A12 [34]. In our study, we found that plasma sRAGE and s100A12 are positively correlated with statistical significance in ACS patients. However, in the stable coronary heart disease, s100 and sRAGE showed a negative correlation, but we failed to observe any statistical significance. S100A12 reflects the degree of inflammatory response in patients with ACS; accordingly, sRAGE works as a protective mechanism with increased secretion over a short period of time.

To further assess the effectiveness of each independent predictor in predicting severe coronary heart disease (high SYNTAX score), we performed a ROC curve analysis. Our results showed that plasma sRAGE and S100A12 exhibited moderate energy in predicting a high SYNTAX score with an AUC of 0.734 and 0.637, respectively. After combining these two factors, the ROC analysis revealed that the area under the ROC curve reached 0.759, which showed a good predictive power for a high SYNTAX score in ACS. This result also further shows that the combination of the two indicators sRAGE and S100A12 has a predictive value for severe coronary heart disease. Several studies have observed that the ratio of AGEs/sRAGE in stable coronary heart disease is positively correlated with the severity of the lesion, but not the patients with acute coronary syndrome as the study object alone, which will lead to different results. Our study reflects the inconsistency of sRAGE expression in different pathological states.

## 5. Conclusion

In conclusion, the plasma levels of sRAGE and S100A12 were increased in patients with ACS; however, these did not change significantly in patients with stable angina and nonstenosis coronary atherosclerosis. The elevated sRAGE concentration may be independently associated with the severity of CAD and the inflammatory process in patients with ACS. sRAGE combined with S100A12 may be used as a predictor of severe coronary heart disease.

## Figures and Tables

**Table 1:** The clinical data for patients.

Patient characteristics	Normal groups (*N* = 61)	NOCA (*N* = 58)	SAP (*N* = 61)	ACS (*N* = 74)	*P*
Age (years)	59.84 ± 8.64	62.45 ± 7.77	64.66 ± 8.97	66.31 ± 9.76	<0.01
Male gender (*n* (%))	32 (52.46%)	42 (72.41%)	44 (72.13%)	53 (71.62%)	0.218
Hypertension (*n* (%))	18 (29.51%)	29 (50.00%)	35 (57.38%)	50 (67.57%)	0.073
Diabetes (*n* (%))	10 (16.39%)	6 (10.34%)	15 (24.59%)	23 (31.08%)	0.028
Smoking (*n* (%))	16 (26.23%)	28 (48.28%)	19 (31.15%)	29 (39.19%)	0.176
BMI (kg/m^2^)	23.95 ± 3.39	25.15 ± 3.57	24.71 ± 3.19	24.18 ± 2.74	0.160
History of medication					
Nitrates (*n* (%))	18 (29.51%)	23 (39.66%)	36 (59.02%)	47 (63.51%)	0.263
Beta blockers (*n* (%))	20 (32.79%)	26 (44.83%)	30 (49.18%)	35 (47.30%)	0.586
ACEIs/ARBs (*n* (%))	8 (13.11%)	14 (24.14%)	12 (19.67%)	13 (17.57%)	0.437
CCBs (*n* (%))	8 (13.11%)	15 (25.86%)	19 (31.15%)	22 (29.73%)	0.161
Aspirin (*n* (%))	16 (26.23%)	21 (36.21%)	52 (85.25%)	72 (97.30%)	0.062
Clopidogrel (*n* (%))	1 (1.64%)	3 (5.17%)	17 (27.87%)	41 (55.41%)	0.024
Statins (*n* (%))	10 (16.39%)	22 (37.93%)	44 (72.13%)	57 (77.03%)	0.031
SBP (mmHg)	118.72 ± 21.76	128.40 ± 18.52	127.93 ± 19.31	129.89 ± 18.53	<0.01
DBP (mmHg)	67.78 ± 12.11	74.26 ± 14.21	74.08 ± 14.03	74.46 ± 17.08	0.031
HR (bpm)	80.39 ± 19.55	76.03 ± 12.51	75.46 ± 11.64	77.99 ± 15.63	0.273
HGB (g/l)	126.39 ± 47.99	127.91 ± 20.51	126.48 ± 23.26	124.07 ± 20.00	0.905
FBS (mmol/l)	5.74 ± 1.92	5.85 ± 2.17	6.45 ± 2.25	6.72 ± 2.88	0.053
HbA1c (%)	3.90 ± 2.62	5.38 ± 0.18	6.61 ± 1.30	6.37 ± 1.13	<0.01
TC (mmol/l)	3.92 ± 1.01	4.41 ± 1.01	4.14 ± 1.29	4.28 ± 1.65	0.185
LDL-C (mmol/l)	2.50 ± 0.80	2.69 ± 0.81	2.59 ± 0.87	2.66 ± 0.83	0.566
TG (mmol/l)	1.53 ± 1.21	1.58 ± 1.42	2.20 ± 6.09	1.50 ± 0.80	0.562
SUA (*μ*mol/l)	344.94 ± 109.93	344.60 ± 117.33	308.83 ± 81.14	330.54 ± 101.61	0.185
Scr (*μ*mol/l)	74.13 ± 18.75	97.47 ± 115.44	83.39 ± 51.18	84.95 ± 45.03	0.474

Abbreviations: BMI: body mass index; ACEIs: angiotensin-converting enzyme inhibitors; ARBs: angiotensin receptor blockers; CCBs: calcium channel blockers; SBP: systolic blood pressure; DBP: diastolic blood pressure; HR: heart rate; HGB: hemoglobin; FBS: fasting blood glucose; HbA1c: glycosylated hemoglobin; TC: total cholesterol; LDL-C: low-density lipoprotein cholesterol; HDL-C: high-density lipoprotein cholesterol; TG: triglyceride; SUA: serum uric acid; Scr: serum creatinine.

## Data Availability

The data used to support the findings of this study are available from the corresponding author upon request.

## Abbreviations

CAD: Coronary artery disease
T2DM: Type 2 diabetes mellitus
AGEs: Advanced glycation end products
RAGE: Receptor for advanced glycation end products
sRAGE: Soluble receptor for advanced glycation end products
MACCE: Long-term major adverse cardiac and cerebrovascular event
NOCA: Nonobstructive coronary atherosclerosis (<50% stenosis in all coronary vessels)
SAP: Stable angina
ACS: Acute coronary syndrome
CAG: Coronary angiography
PCI: Percutaneous coronary intervention
pPCI: Primary percutaneous coronary intervention
NSTE-ACS: Non-ST-elevation acute coronary syndrome
NSTEMI: Non-ST segment elevation myocardial infarction
SS: SYNTAX score
ROC: Receiver operating characteristic.
